# Morbidity profile of cotton mill workers

**DOI:** 10.4103/0019-5278.75697

**Published:** 2010

**Authors:** Pravin N. Yerpude, Keerti S. Jogdand

**Affiliations:** Department of Community Medicine, Katuri Medical College and Hospital, Guntur 522 019, Andhra Pradesh, India

**Keywords:** Cotton mill workers, morbidity profile

## Abstract

**Objective:**

To study the morbidity pattern among cotton mill workers.

**Material and Methods:**

This cross-sectional study was conducted in cotton mills in Guntur District (AP) in January 2009 to May 2009. Total 474 workers were included in the study.

**Results:**

All study subjects were male. Most of the study subjects belonged to age group 30–40 years (56.96%) and lower socioeconomic status (36.09%) according to modified Kuppuswamy’s classification. The literacy status was varied with 5.70% being illiterate and 37.13% were educated up to primary school. Most of workers were working in Ring frame (41.56%) and majority (58.44%) were working for the last 5–10 years. Mean height of study subjects was 147.42 cm and mean weight was 55.11 kg. The common morbid conditions found were eosinophilia (18.35%), iron deficiency anemia (28.90%), byssinosis grade 1 (7.80%), dental stains (6.54%), refractive errors (7.80%), chronic bronchitis (4.85%), and upper respiratory tract infection (8.64%).

## INTRODUCTION

Occupational health is a branch of community medicine which deals with the effects of occupation or workplace on human health.[1] Every occupation is associated with one or other ill effects on health. One such occupational group is cotton mill workers. Cotton mill workers are susceptible to various morbid conditions by virtue of workplace and working conditions. These morbid conditions may range from chronic respiratory diseases due to cotton dust inhalation[2] to anemia because of nutritional deficiency. Though many studies on chronic respiratory disease among cotton mill workers have been carried out,[3–5] a study including complete health profile of cotton mill workers is limited. With this background, the present study was undertaken to study the morbidity profile among cotton mill workers

## MATERIALS AND METHODS

The present cross-sectional study was carried out among cotton mill workers in Guntur District (AP) from January 2009 to May 2009. All 474 workers who were working in the cotton mill were included in the study. Interview technique was used to collect information on a predesigned pro forma regarding demographic data, occupational history, and history of present and past complaints. This was followed by complete clinical examination, laboratory investigations such as hemoglobin estimation, peripheral smear examination, and chest radiograph of each study subjects. Standard diagnostic criteria were used for the diagnosis of various morbid conditions.[6–8]

For byssinosis Roach and Schilling criteria[2] was used.

## RESULTS

Distribution of study subjects according to demographic characteristic is shown in Table 1. Majority of study subjects belonged to 30–35 years (31.22%) and 35–40 years (25.74%) age group. 37.13% workers had primary education while 28.90% workers had educational attainment up to middle school level. Majority of study subjects belonged to lower (36.09%) and upper lower (34.38%) socioeconomic status according to modified Kuppuswamy’s socioeconomic status scale.

Table 2 shows the distribution of study subjects according to occupational and personal characteristics. Majority (58.44%) of workers had considerable duration of exposure, i.e., 5–10 years and that too in dusty departments such as winding, ring frame, speed frame, and carding. Of the total 474 study subjects, only 20.46% were smokers.

Distribution of study subjects according to anthropometric characteristics is shown in Table 3. The mean height of study subjects was 147.42 cm. Majority (36.50%) of study subjects had height between 160 and 165 cm. Similarly, the mean weight of study subjects was 55.11 kg with majority (38.61%) of study subjects having body weight between 55 and 65 kg. Using body mass index as a parameter for obesity, 37.34% study subjects were found to have obesity of varying degrees.

Table 4 shows the various morbid conditions among workers. Most common morbid conditions included eosinophilia (18.35%), iron deficiency anemia (28.90%), byssinosis grade 1 (7.80%), dental stains (6.54%), refractive errors (7.80%), chronic bronchitis (4.85%), upper respiratory tract infection (8.64%).

**Table 1:** Distribution of study subjects according to demographic characteristics

Characteristics	No. (%)
Age (years)	
<25	38 (8.02)
25–30	82 (17.30)
30–35	148 (31.22)
35–40	122 (25.74)
40–45	61 (12.87)
>45	23 (4.85)
Education	
Illiterate	27 (5.70)
Primary	176 (37.13)
Middle school	137 (28.90)
Secondary	67 (14.13)
Higher secondary	27 (5.70)
Graduate and above	40 (8.44)
Socioeconomic status	
Upper	23 (4.85)
Upper middle	34 (7.17)
Lower middle	83 (17.51)
Upper lower	163 (34.38)
Lower	171 (36.09)

**Table 2:** Distribution of study subjects according to occupational and personal characteristic

Characteristics	No. (%)
Duration of exposure (years)	
<5	67 (14.14)
5–10	277 (58.44)
>10	130 (27.42)
Department	
RJK winding	137 (28.90)
Speed frame	24 (5.06)
Ring frame	197 (41.56)
Blow room	03 (0.63)
Carding	17 (3.59)
Others	96 (20.26)
Smoking	
Smokers	97 (20.46)
Nonsmokers	377 (79.54)

**Table 3:** Distribution of study subjects according to anthropometric characteristics

Characteristics	No. (%)
Height (cm)	
<150	03 (0.63)
150–155	13 (2.74)
155–160	67 (14.14)
160–165	173 (36.50)
165–170	127 (26.79)
>170	91 (19.20)
Weight (kg)	
>45	07 (1.48)
45–55	157 (33.12)
55–65	183 (38.61)
65–75	73 (15.40)
>75	54 (11.39)
BMI	
<25	297 (62.66)
25–29.9	83 (17.51)
30–39.9	79 (16.67)
>40	15 (3.16)

**Table 4:** Distribution of study subjects according to morbid conditions

Morbid conditions (ICD no.)	No. (%)
Acid peptic disease (T30.4)	08 (1.68)
Amoebiasis (A06.9)	17 (3.58)
Allergic rhinitis (J30.4)	05 (1.05)
Apthous ulcer (K12.0)	10 (2.10)
Byssinosis (J66.0)	37 (7.80)
Bronchial asthma (J45.9)	08 (1.68)
Chronic bronchitis (J44.8)	23 (4.85)
Dental stains (KO3.6)	31 (6.54)
Dental caries (K02.9)	27 (5.69)
Eosinophilia (D72.1)	87 (18.35)
Hypertension (I10)	14 (2.95)
Injury (T14.9)	13 (2.75)
Iron deficiency anemia (D50.9)	137 (28.90)
Refractive errors (H52.7)	37 (7.80)
Tuberculosis (A16.2)	04 (0.84)
Upper respiratory infections (JO6.9)	41 (8.64)

**Table:** Roach and Schilling criteria for grading byssinosis

Grade 0	No symptoms of chest tightness or breathlessness on Monday
Grade ½	Occasional chest tightness on Mondays or mild symptoms such as irritation of the respiratory tract on Monday
Grade 1	Chest tightness and/or breathlessness on Mondays only
Grade 2	Chest tightness and/or breathlessness on Mondays and other days
Grade 3	Grade 2 symptoms accompanied by evidence of permanent respiratory disability from reduced ventilatory capacity.

## DISCUSSION

In the present study, a respiratory morbidity is due to the inflammatory and allergic response to inhaled cotton dust particles resulting in chronic bronchitis, bronchial asthma, upper respiratory tract infection, and byssinosis. Other studies have also reported similar findings.[3–5] Allergic response to inhaled cotton dust particles may have been responsible for eosinophilia. High prevalence of dental problems suggests a poor oral hygiene among study subjects. As most of the study subjects belonged to upper lower and lower socioeconomic class, they have poor purchasing capacity, which may be responsible for a high prevalence of iron deficiency anemia among study subjects.

## CONCLUSION

The present study indicates a need of health education regarding use of personal protective devices such as mask and other respiratory devices and maintaining high standard of oral and personal hygiene. Tobacco smoking and chewing should be prevented.
